# cFOS expression in the prefrontal cortex correlates with altered cerebral metabolism in developing germ-free mice

**DOI:** 10.3389/fnmol.2023.1155620

**Published:** 2023-04-20

**Authors:** Trinity Pate, Daniel C. Anthony, Daniel E. Radford-Smith

**Affiliations:** Department of Pharmacology, University of Oxford, Oxford, United Kingdom

**Keywords:** microbiota, cFOS, short-chain fatty acids (SCFAs), germ free animal, metabolomics, acetate, glutamate, glutamine

## Abstract

**Introduction:**

The microbiota plays a critical role in modulating various aspects of host physiology, particularly through the microbiota-gut-brain (MGB) axis. However, the mechanisms that transduce and affect gut-to-brain communication are still not well understood. Recent studies have demonstrated that dysbiosis of the microbiome is associated with anxiety and depressive symptoms, which are common complications of metabolic syndrome. Germ-free (GF) animal models offer a valuable tool for studying the causal effects of microbiota on the host.

**Methods:**

We employed gene expression and nuclear magnetic resonance (NMR)-based metabolomic techniques to investigate the relationships between brain plasticity and immune gene expression, peripheral immunity, and cerebral and liver metabolism in GF and specific pathogen-free (SPF) mice.

**Results:**

Our principal findings revealed that brain acetate (*p* = 0.012) was significantly reduced in GF relative to SPF mice, whereas glutamate (*p* = 0.0013), glutamine (*p* = 0.0006), and N-acetyl aspartate (*p* = 0.0046) metabolites were increased. Notably, *cFOS* mRNA expression, which was significantly decreased in the prefrontal cortex of GF mice relative to SPF mice (*p* = 0.044), correlated with the abundance of a number of key brain metabolites altered by the GF phenotype, including glutamate and glutamine.

**Discussion:**

These results highlight the connection between the GF phenotype, altered brain metabolism, and immediate-early gene expression. The study provides insight into potential mechanisms by which microbiota can regulate neurotransmission through modulation of the host’s brain and liver metabolome, which may have implications for stress-related psychiatric disorders such as anxiety.

## Introduction

The gut microbiome is a complex and dense microbial network that regulates many aspects of human physiology and behavior. In adulthood, it is estimated that there are over 1,000 different species comprising approximately 10^14^ organisms, although inter-individual variation is significant (Qin et al., 2010).

This postnatal colonization and persistence of microbiota throughout life is a normal part of mammalian development, with microbiota metabolism contributing to the function of several host systems and processes including host inflammatory responses, energy metabolism, and gastrointestinal absorption (Cebra, 1999; Velagapudi et al., 2010; Semova et al., 2012). The gut microbiota digest host-indigestible dietary fibers, producing metabolic by-products which are absorbed and utilized by the host such as short-chain fatty acids (SCFAs), which regulate several aspects of host physiology (van der Hee and Wells, 2021). This exemplifies the symbiotic relationship between bacteria and their host organisms, and highlights the critical role of microbiota in host metabolism.

Gut microbiota metabolism has also been shown to modify brain chemistry and behavior via the bidirectional MGB axis (Cryan et al., 2019). For instance, it has been demonstrated that microbiota are required for normal social development in mice (Barreau et al., 2004; Zheng et al., 2016). Metabolic profiling studies have even shown that the maternal microbiome can modulate fetal neurodevelopment in mice (Bravo et al., 2011). Rodent models of maternal separation demonstrate how early life stress modifies the composition of the gut flora (Sobko et al., 2006) and can result in long-term changes in gut physiology, including the permeability of the epithelial barrier which can trigger exaggerated immune responses (Nicolas et al., 2022). Gut dysbiosis also affects brain chemistry and behavior. Germ-free mice colonized by faucal microbiota transplant with “major depressive disorder (MDD) microbiota,” but not healthy control microbiota, had disturbed serum and hippocampal metabolomes and begin displaying anxiety- and depressive-like behaviors (Schéle et al., 2013).

Germ-free (GF) mice lack microbiota from birth and have proved essential in understanding the casual impact of microbiota on host physiology. In the brain, GF mice display an immature microglial phenotype with altered morphological characteristics and gene expression profiles, which can be restored by engraftment of a complex gut microbiota, or short-chain fatty acid (SCFA) supplementation (Erny et al., 2015). Microglia are the resident macrophages of the central nervous system (CNS), responding to brain infections and coordinating downstream immune reactions (Borst et al., 2021), whilst also regulating synaptic pruning and shaping neural circuits in the developing brain (Schafer et al., 2012). Their deficiency in GF mice may partly explain their altered brain chemistry and immune activity.

Recent reviews have introduced the concept of the “gut-liver-brain axis,” which emphasizes the central role of the liver in peripheral metabolism and immune regulation (Tripathi et al., 2018; Brescia and Rescigno, 2021; Kronsten et al., 2022). Extensive inter-organ communication exists between the liver and the gut, particularly at the level of circulating metabolites, through the portal vein. Moreover, hepatic encephalopathy is a severe yet common complication of end-stage liver disease, whereby neurotoxic metabolites (some gut-derived) are not able to metabolized by the liver. Despite the increasing awareness of the gut-liver-brain axis in health and disease, the liver and brain metabolome have not been jointly analyzed in GF and SPF mice.

Current understanding of GF physiology is still relatively limited. Most studies have focused on gene expression, far fewer have identified metabolomic changes, and to our knowledge, no study has combined both techniques in the same animal and drawn inferences between them. Global metabolite profiling, otherwise known as untargeted metabolomics, can be used to explore metabolic differences in phenotypes as a consequence of the microbiome. Exploring the gene expression changes in conjunction with metabolomic changes could identify novel pathways relating to the microbial modulation of brain function. Therefore, the aim of the current study was to interrogate the contribution of the gut microbiota to shaping the relationship between neuroplasticity gene expression in the prefrontal cortex (PFC) and cerebral metabolite profiling. This is supported by previous reports of altered metabolism in the cerebral cortex of GF mice (Lai et al., 2021), and the relationship between early life probiotic supplementation, cerebral metabolite levels, and altered PFC gene expression (Radford-Smith et al., 2022). Accordingly, we hypothesized that GF status would affect cerebral metabolite levels through altered neurodevelopmental trajectories, with the understanding that the maternal generation was also germ free and microbial metabolites are thought to play a role in normal brain development (Vuong et al., 2020). We also expected to discover mRNA-metabolite correlations in SPF mice that dissociated in GF mice which would aid our understanding of the relationship between microbiota, metabolites, and brain function. An examination of the biochemical pathways perturbed in GF mice will provide further insights into the role of the gut microbiota in brain metabolism and may be indicative of future therapeutic avenues to pursue.

## Materials and methods

### Animals

**FIGURE 1:**
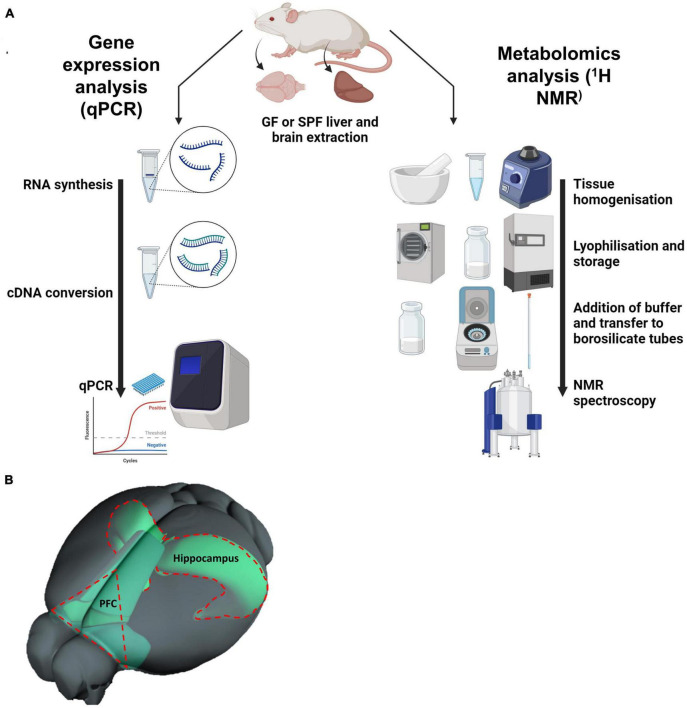
**(A)** Diagram depicting the methodology of the project: quantitative PCR (qPCR) and 1D ^1^H nuclear magnetic resonance (NMR) spectroscopy. **(B)** Illustration of prefrontal cortex within the mouse brain, relative to the hippocampus. **(B)** Is adapted from the Allen Mouse Brain Connectivity Atlas.

C57Bl/6 mice were killed by cervical dislocation and the whole brain and liver were immediately dissected from juvenile (3-week-old) female GF and SPF was animals and snap frozen in isopentane. Mice were interrogated for liver and brain qPCR and metabolomic analyses (Figure 1). A total of 2 cohorts of mice were used in this experiment. For the liver gene expression analysis, 5 SPF and 7 GF mice were used. For the liver and brain metabolomics analysis, and brain qPCR analysis, a separate cohort of 6 SPF and 6 GF mice were used.

### Gene expression

#### RNA extraction and cDNA conversion

The prefrontal cortex was dissected from the whole brain by making a V-shaped incision at the most anterior part of the brain, avoiding the olfactory bulb and white matter. The Qiagen RNeasy Mini Kit (Qiagen Ltd., Manchester, UK) was utilized for RNA extraction, using methods described in detail elsewhere (Radford-Smith et al., 2022). A total of 1,000 nanograms of RNA was converted to cDNA using the high-capacity cDNA reverse transcription kit (Thermo Fisher, Loughborough, UK). To assess suitability for cDNA conversion, both the total RNA and RNA purity were assessed using a NanoDrop1000 (Thermo Fisher, Loughborough, UK).

#### Quantitative (q)PCR

Quantitative PCR was conducted using a LightCycler^®^ 480 instrument (Roche Diagnostics, West Sussex, UK) with SYBR green master mix (Primerdesign, Hampshire, UK). 25 ng cDNA was used per 10 uL reaction. Primers (Table 1) were obtained from Primerdesign, Merck Life Science LTD (Dorset, UK), or Bio-Rad Laboratories (Hertfordshire, UK) and used at a working concentration of 300 nM. *GAPDH* was used as the reference gene in both the brain and liver tissue. Melt curves were analyzed to confirm that a singular PCR product had been generated for each reaction. Samples were run in duplicate and normalized using 2^–Δ^ *^Ct^* for each sample, where ΔCt = (Ct_target_ gene–Ct_reference_ gene), and then analyzed data as fold change in gene expression relative to the control group.

**TABLE 1:** List of primers for qPCR.

Gene	Forward sequence (5′ to 3′)	Reverse sequence (5′ to 3′)	Tissue	References
*GAPDH*	AACGACCCCTTCATTGAC	TCCACGACATACTCAGCAC	Brain/Liver	PrimerDesign
*TNF*	GCCTCCCTCTCATCAGTTCTAT	TTTGCTACGACGTGGGCTA	Brain/Liver	PrimerDesign
*IL-1B*	CAACCAACAAGTGATATTCTCCAT	GGGTGTGCCGTCTTTCATTA	Brain/Liver	PrimerDesign
*IL-6*	TCCATCCAGTTGCCTTCTTG	GGTCTGTTGGGAGTGGTATC	Brain/Liver	PrimerDesign
*TLR4*	CTGGCTAGGACTCTGATCATG	GCATTGGTAGGTAATATTAGGAACTA	Brain/Liver	Strekalova et al., 2015
*SAA-2*	TGGCTGGAAAGATGGAGACAA	AAAGCTCTCTCTTGCATCACTG	Liver	PrimerDesign
*BDNF*	GGATATTGCGAAGGGTTATTAGATT	GGAAGGTAATGTGTCTTGTTTGAA	Brain	PrimerDesign
*5-HT_1A_*	AACCAGTTTTGTGTCCTCTCA	AGCACCTAAATAATTTTCTTCTCTGA	Brain	PrimerDesign
*5-HT_2A_*	CAGGCAAGTCACAGGATAGC	TTAAGCAGAAAGAAAATCCCACAG	Brain	Hebert et al., 2021
*5-HT_6_*	CCTGACCCTCGGCATCCTG	GGTTCATGGTGCTATTACAGTATCC	Brain	PrimerDesign
*GLUN1*	CCAGACTAAAGATAGTGACAA	ACCATTGACTGTGAACTC	Brain	PrimerDesign
*GLUN2A*	GCTTTCCTTGAACCCTTCAG	GGGGAGCTTTCCCTTTGGCTAAGTT	Brain	Hebert et al., 2021
*GLUN2B*	TTGGTGAGGTGGTCATGAAG	GGCTCTAAGAAGGCAGAAGGT	Brain	Hebert et al., 2021
*GLUN2C*	GGTTGCCATCACTGTCTTCA	CCACACGGACTTGCCAAT	Brain	PrimerDesign
*CREB1*	AGTGACTGAGGAGCTTGTACCA	TGTGGCTGGGCTGAAC	Brain	Iñiguez et al., 2021
*GSK3B*	GACAAGCATTTAAGAACCGAGA	ACCAGGTAAGGTAGACCTACATC	Brain	Iñiguez et al., 2021
*PSD-95*	TCTGTGCGAGAGGTAGCAGA	AAGCACTCCGTGAACTCCTG	Brain	Zhao et al., 2021
*SYP*	BioRad unique assay ID: qMmuCID0023269		Brain	BioRad
*ATP1A2*	BioRad unique assay ID: qMmuCID0024259		Brain	BioRad
*PFKFB3*	BioRad unique assay ID: qMmuCID0014746		Brain	BioRad
*cFOS*	AACCGCATGGAGTGTGTTGTTCC	TCAGACCACCTCGACAATGCATGA	Brain	Iñiguez et al., 2021
*ΔFOSB*	AGGCAGAGCTGGAGTCGGAGAT	GCCGAGGACTTGAACTTCACTCG	Brain	Iñiguez et al., 2021
*ZIF-268*	TCGGCTCCTTTCCTCACTCA	CTCATAGGGTTGTTCGCTCGG	Brain	Iñiguez et al., 2021
*GFAP*	GCGCTCAATGCTGGCTTCA	TCTGCCTCCAGCCTCAGGTT	Brain/Liver	Borghi et al., 2019

### Metabolic profiling

#### Sample preparation

Samples were prepared in small batches to minimize variation, using an optimized protocol based on previously published methods (Waters et al., 2001). Between 115 and 135 mg of fresh liver or brain (left hemisphere of cerebrum) were homogenized with a pestle and mortar on dry ice; equipment was dry-wiped between samples to prevent cross-contamination. Samples were diluted eight-fold (μL/mg) in 50% acetonitrile in pure water and further homogenized by being briefly vortexed. Samples were then centrifuged at 5,060 × *g* for 5 min at 4°C and 750 μL of each sample was aspirated into a fresh Eppendorf. Samples were then lyophilized and stored at −80°C until the day of NMR analysis where they were resuspended by vortex in 600 μL of 75 mM sodium phosphate buffer (5:1 disodium phosphate [Na2HPO4] and monosodium phosphate [NaH2PO4] in 100% D2O, pH = 7.4) and centrifuged at 2,500 × *g* for 5 min at 4°C to remove any particulate matter. Lastly, samples were transferred to a 5 mm borosilicate tube using a glass Pasteur pipette, ready for NMR analysis.

#### 1D ^1^H nuclear magnetic resonance (NMR) spectroscopy and data processing

^1^H NMR spectra were obtained using a 700-MHz Bruker AVII spectrometer, operating at a 16.4T equipped with a ^1^H (13C/15N) TCI cryoprobe. Sample temperature was maintained at 298K, and spectra were acquired using a 1D NOESY experiment (brain) and CPMG experiment (liver) as described previously (Radford-Smith et al., 2022). Topspin 2.1 (Bruker, Germany) was used for data processing: to manually phase the spectra, correct baselines, and reference the chemical shifts to the lactate CH_3_ doublet resonance at δ = 1.33 ppm. All spectra of the same tissue were overlayed and automatically binned with 0.02 ppm-sized bins, although some compounds were manually binned. Each bin was normalized to the sum of all integrals in the spectrum of each sample and data exported to R v3.3.1 and Microsoft Excel for statistical analysis and metabolite identification. Metabolites were assigned by comparing spectral peaks to the Human Metabolome Database (Wishart et al., 2018) and by spiking in known compounds.

### Statistical analysis

Statistical analysis was performed using GraphPad Prism 6 and R v3.3.1 (R Foundation for Statistical Computing). An in-house R script was used for principal component analysis (PCA) of pareto-scaled data to initially visualize and inspect the data. PCA loadings were plotted to identify key metabolites in the liver and brain. Individual metabolites were then run through the following analyses.

Initially, Grubb’s test for outliers was performed on each group for all qPCR and metabolomic data and statistically significant outliers (*p* < 0.05) were removed from downstream analyses. Normality of data was assessed using the Kolmogorov–Smirnov (KS) test: if assumed (*p* < 0.1), a parametric independent samples *t*-test was conducted, if not assumed (*p* < 0.1, or the test concluded N was too small due to exclusion of anomalous results) then the non-parametric Mann–Whitney U test was conducted. For parametric data, the independent samples t-test was followed by an *F*-test to compare variances (*p* < 0.05) and if significant, the *t*-test was repeated using Welch’s correction to account for the unequal variances between the populations. Results from *t*-tests and Mann–Whitney U tests were considered statistically significant if *p* < 0.05 and trending if *p* < 0.1. Data were then visualized as boxplots where the upper and lower bounds of the box denote the interquartile range, the line within the box as the median, and whiskers encompassing the full range of values.

Correlations between brain qPCR and all metabolome data were performed using the Spearman rank correlation. Since normality could not be assumed due to too low an N number for correlation analysis, a non-parametric test was used. False discovery rate (FDR) analysis was carried out to correct for multiple testing and significant correlations (*q* < 0.05) were visualized as scatterplots.

## Results

### Germ-free mice have altered cerebral and liver metabolite profiles

**FIGURE 2:**
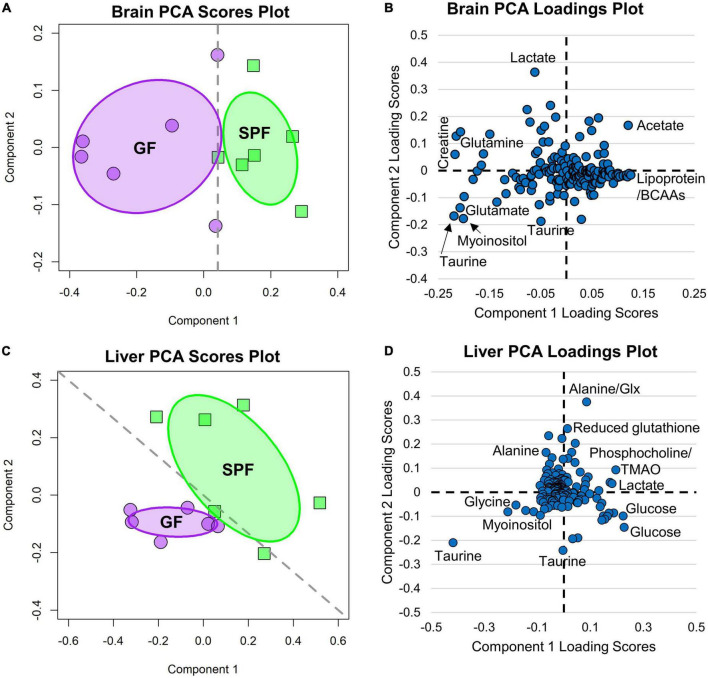
PCA score plots comparing the GF and SPF liver and brain metabolome. Purple data points are from GF (*n* = 6) and green from SPF (*n* = 6) mice. **(A)** Brain PCA plot; P1 = 71%, P2 = 11% and **(B)** corresponding loadings plot. **(C)** Liver PCA plot; P1 = 43%, P2 = 22% and **(D)** corresponding loadings plot.

**FIGURE 3:**
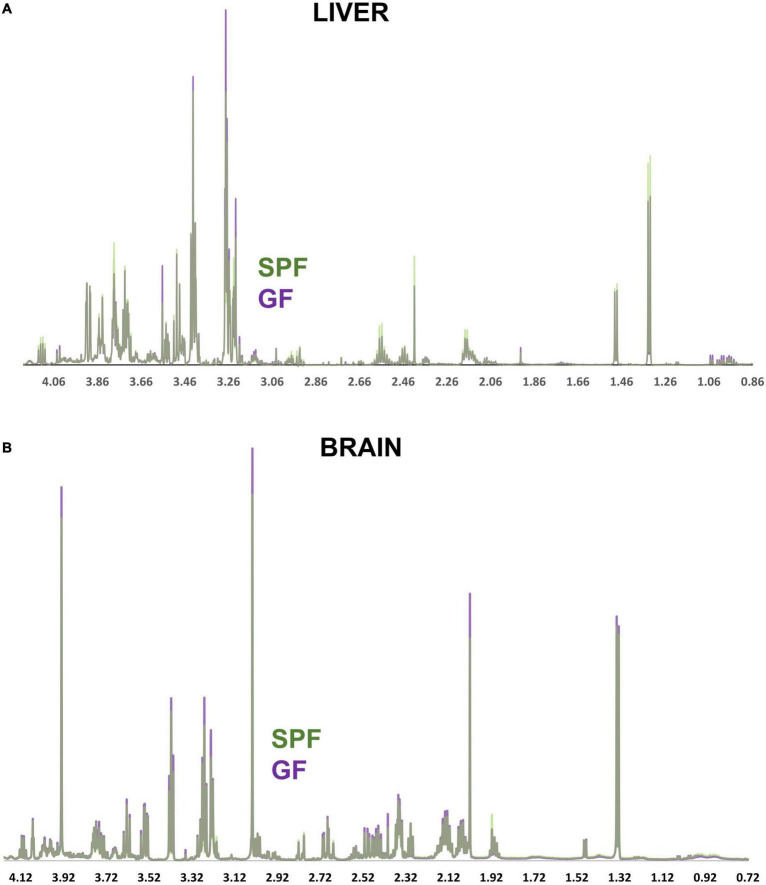
NMR spectra comparing the GF and SPF liver and brain metabolome. **(A)** Median spectral intensity of the GF (purple, *n* = 6) and SPF (green, *n* = 6) liver metabolome. **(B)** Median spectral intensity of the GF (purple, *n* = 6) and SPF (green, *n* = 6) brain metabolome. Several metabolites were altered by absence of bacterial flora in both liver and brain GF tissue (Figures 4, 5). In the brain, taurine, myoinositol, creatine, glutamate, glutamine, choline, and NAA were significantly increased in GF mice relative to SPF animals (Figure 4). Conversely, acetate and various lipoprotein resonances were reduced in GF animals (Figure 4). Inspection of the corresponding loadings plot indicates that cerebral lactate and taurine levels may have contributed to the variation observed in the GF mice brain samples (Figure 2B).

To study the impact of the microbiome on mouse metabolite profiles, untargeted proton NMR spectroscopy was performed on liver and cerebrum tissue extracts of female, 3-week-old GF and SPF mice. In both tissues, there was significant variation between the two groups, visualized in the PCA scores plots (Figures 2A, C) and NMR spectra (Figure 3). PCA loadings plots (Figures 2B, D) were then used to determine which metabolites were contributing most to the group variation (Table 2).

**TABLE 2:** Summary of metabolic changes in the brain and liver of germ free mice.

Metabolite	Organ	Direction in GF mice	Primary function(s)
Taurine	Brain	↑	Brain neurotransmitter
Myoinositol	Brain	↑	Second messenger precursor, brain osmolyte
Creatine	Brain	↑	Energy metabolism, ATP generation
Glutamine	Brain	↑	Neurotransmitter precursor to Glutamate and GABA
Glutamate	Brain	↑	Primary excitatory neurotransmitter, metabolic precursor to GABA
NAA	Brain	↑	Brain osmolyte, possible neurotransmitter
Lipids	Brain	↓	Structural (cell membrane component)
Acetate	Brain	↓	Energy metabolite, putative signaling molecule for microglia maturation
GABA	Brain	↓	Primary inhibitory neurotransmitter
Lipoprotein	Brain	↓	Lipid trafficking and energy metabolism
Choline/Phosphocholine	Brain and Liver	↑ and ↓	Neurotransmitter precursor for acetylcholine, structural component of cell membrane (as phospholipid), methyl donor (epigenetic regulation)
BCAAs (e.g., Leucine)	Brain and Liver	↓ and ↑	Protein synthesis and turnover, neurotransmitter production (nitrogen donor)
Phosphatidylcholine	Liver	↑	Phospholipid (key component of cell membranes)
Glycine	Liver	↑	Protein synthesis (key component of collagen)
Aromatic amino acids (histidine, phenylalanine)	Liver	↑	Phenylalanine is an important molecular precursor for catecholamines, histidine is a precursor of histamine
Succinate	Liver	↓	Energy metabolism
TMAO	Liver	↓	Microbial metabolite derivative
Reduced glutathione	Liver	↓	Antioxidant

**FIGURE 4:**
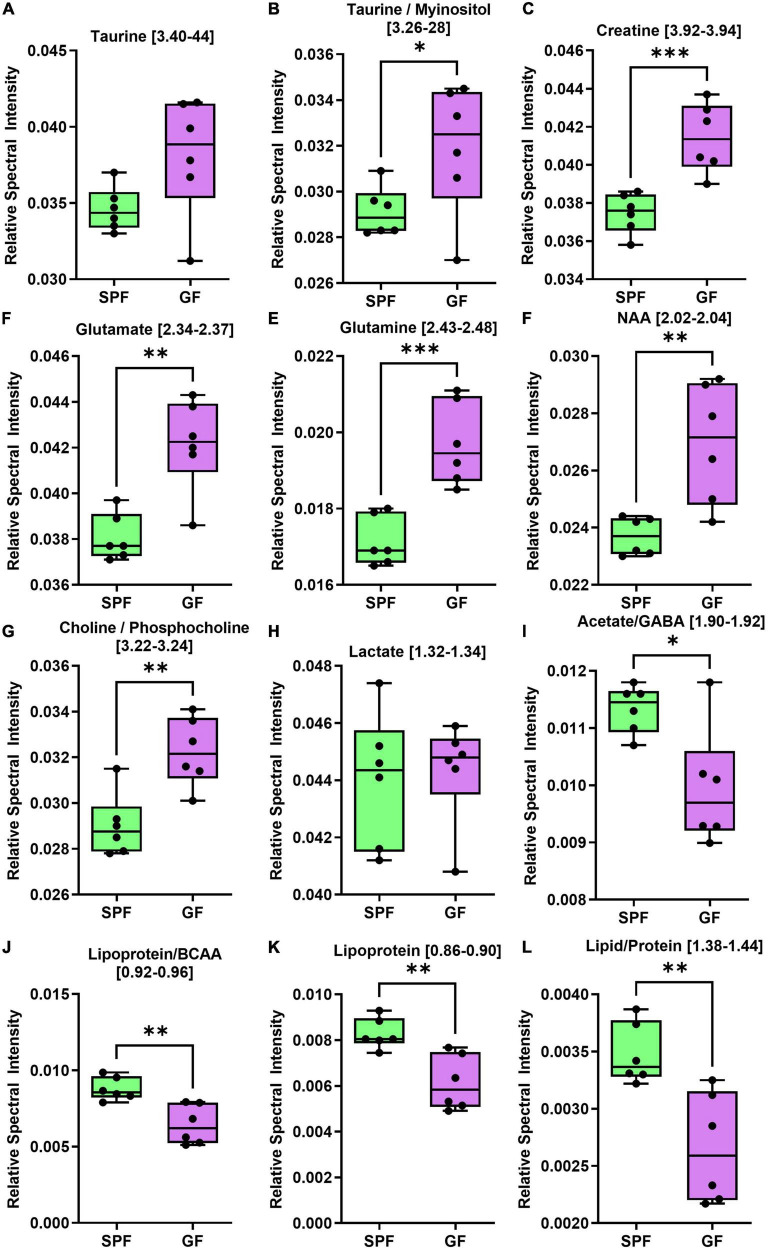
The brain (cerebrum) metabolome is altered in GF (*n* = 6) compared to SPF (*n* = 6) mice. Boxplots represent the interquartile range (IQR), and whiskers display minimum/maximum values. **(A)** Taurine was increased in GF (*p* = 0.0649). **(B)** Taurine/myoinositol (*p* = 0.0481*), **(C)** creatine (*p* = 0.0010^***^), **(D)** glutamate (*p* = 0.0013^**^), **(E)** glutamine (*p* = 0.0006*), **(F)** NAA (*p* = 0.0046^**^), and **(G)** choline/phosphocholine (*p* = 0.0028^**^) were all significantly increased in GF. **(H)** There were no significant variations in lactate (*p* = 0.7975). **(I)** Acetate/GABA (*p* = 0.0120*), **(J)** lipoprotein/BCAA (*p* = 0.0031^**^), **(K)** lipoprotein (*p* = 0.0033^**^), and **(L)** lipid/protein (*p* = 0.0043^**^) were all significantly lower in GF. In the liver, several amino acids were increased in GF mice relative to SPF controls (Figure 5), whereas certain microbial metabolites (succinate, TMAO) were significantly reduced in relative abundance.

**FIGURE 5:**
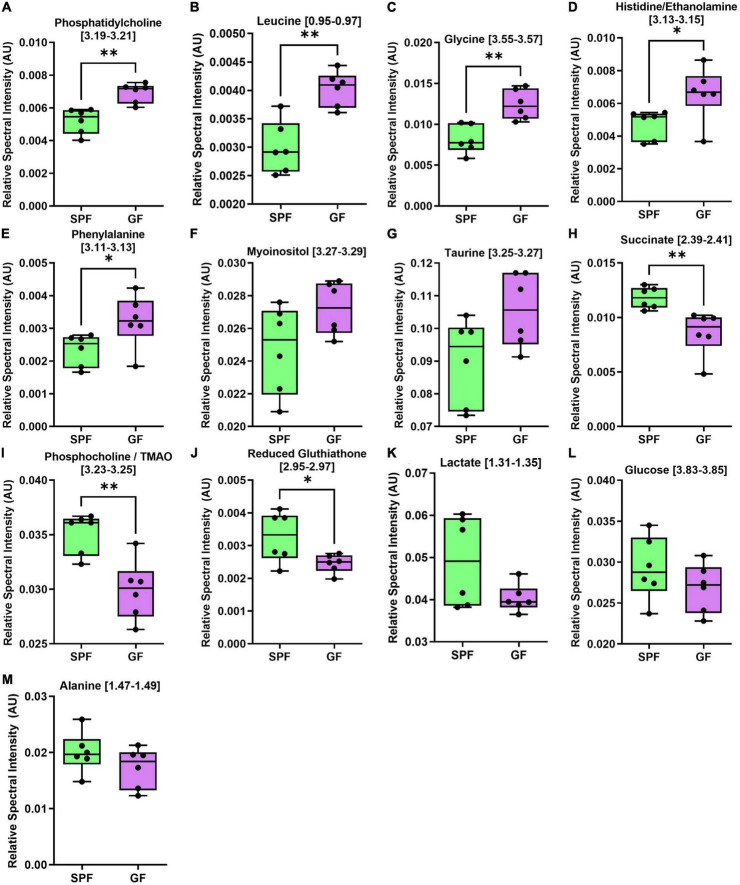
The liver metabolome is altered in GF (*n* = 7) compared to SPF (*n* = 5) mice. Boxplots represent the interquartile range (IQR), and whiskers display minimum/maximum values. **(A)** Phosphatidylcholine (*p* = 0.0015^**^), **(B)** leucine (*p* = 0.0010^**^), **(C)** glycine (*p* = 0.0019^**^), **(D)** histidine/ethanolamine (*p* = 0.0327*), and **(E)** phenylalanine (*p* = 0.0451*) were all significantly increased in GF. **(F)** Myoinositol (*p* = 0.0532) and **(G)** taurine (*p* = 0.0812) were increased in GF. **(H)** Succinate (*p* = 0.0057^**^), **(I)** phosphocholine/TMAO (*p* = 0.0030^**^), and **(J)** reduced glutathione (*p* = 0.0499*) were significantly decreased in GF. **(K)** Lactate (*p* = 0.1012), **(L)** glucose (*p* = 0.2489), and **(M)** alanine (*p* = 0.2156) were not significantly different between groups.

Several metabolites were altered by absence of bacterial flora in both liver and brain GF tissue (Figures 4, 5). In the brain, taurine, myoinositol, creatine, glutamate, glutamine, choline, and NAA were significantly increased in GF mice relative to SPF animals (Figure 4). Conversely, acetate and various lipoprotein resonances were reduced in GF animals (Figure 4). Inspection of the corresponding loadings plot indicates that cerebral lactate and taurine levels may have contributed to the variation observed in the GF mice brain samples (Figures 2A, B).

In the liver, several amino acids were increased in GF mice relative to SPF controls (Figure 5), whereas certain microbial metabolites (succinate, TMAO) were significantly reduced in relative abundance.

### Germ-free mice have significant changes in gene expression in brain and liver tissue

**FIGURE 6:**
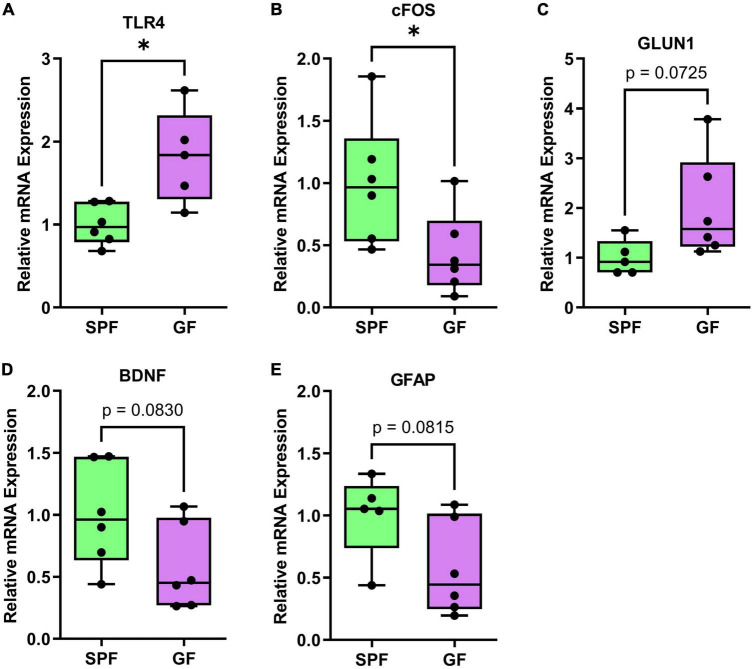
Significant or trending genes from brain qPCR analysis in GF (*n* = 6) and SPF (*n* = 6) mice. *p < 0.05, trending *p* < 0.1. Boxplots represent the interquartile range (IQR) and whisker display minimum/maximum values. **(A)** TLR4 had significantly higher expression in GF (*p* = 0.0101*). **(B)** cFOS had significantly lower expression (*p* = 0.0435*). **(C)** GLUN1 (*p* = 0.0725) had higher expression, **(D)** BDNF (*p* = 0.0830) and **(E)** GFAP (*p* = 0.0815) had lower expression.

**FIGURE 7:**
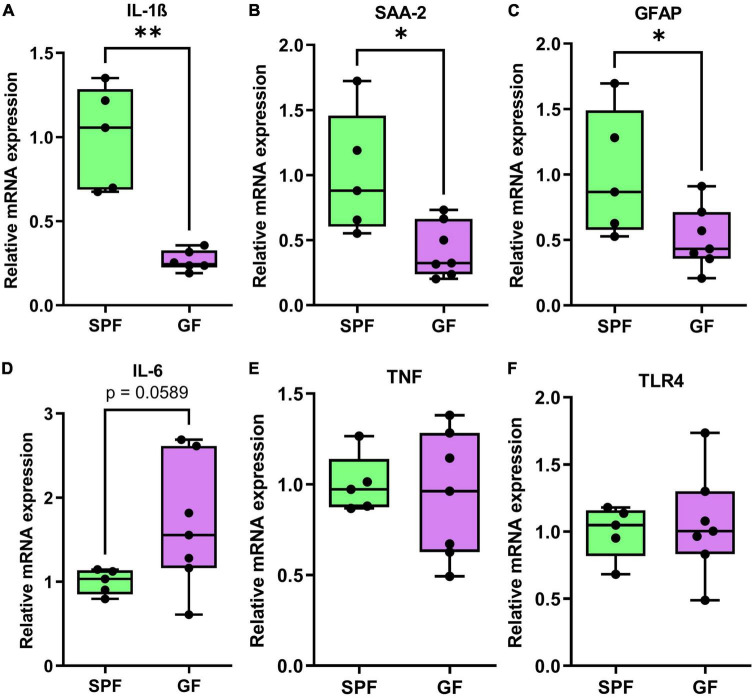
qPCR genes analysis of liver tissue in GF (*n* = 7) and SPF (*n* = 5) mice. Boxplots represent the interquartile range (IQR) and whiskers display minimum/maximum values. **(A)** Interleukin-1ß (*p* = 0.0050^**^), **(B)** serum amyloid A (*p* = 0.0162*), and **(C)** glial fibrillary acidic protein (*p* = 0.0424*) were all significantly lower in GF. **(D)** Interleukin-6 (*p* = 0.0589) was trending higher in GF animals relative to SPF animals. **(E)** TNF (*p* = 0.7184) and **(F)** toll-like receptor-4 (*p* = 0.7961) were not significantly different between GF and SPF animals.

In the frontal cortex of GF and SPF mice, we profiled the mRNA expression of 22 genes (Table 1) relating to neuroplasticity, immunity, and serotonin and glutamate receptors (Figure 6 and Supplementary Figure 1). In the liver, 6 genes were investigated, including *IL-1B, SAA2, GFAP, IL-6, TNF, and TLR4* (Figure 7 and Table 1).

In the prefrontal cortex, *TLR4* was increased in GF mice relative to SPF controls (*p* = 0.01), whereas *cFOS* was decreased (*p* = 0.044). *BDNF* (*p* = 0.083) and *GLUN1* (*p* = 0.073) showed a numeric (non-significant) reduction and increase in GF mice, respectively.

In the liver, mRNA expression of immunity-related factors *IL-1B* (*p* = 0.0050), *SAA2* (*p* = 0.016), and *GFAP* (*p* = 0.042) were reduced in GF mice relative to SPF controls (Figure 6). *IL-6* (*p* = 0.0589) was trending higher in GF animals, but there was no difference in mRNA levels of *TNF* or *TLR4* in the liver (Figure 7).

### Correlations between metabolites and gene expression

**FIGURE 8:**
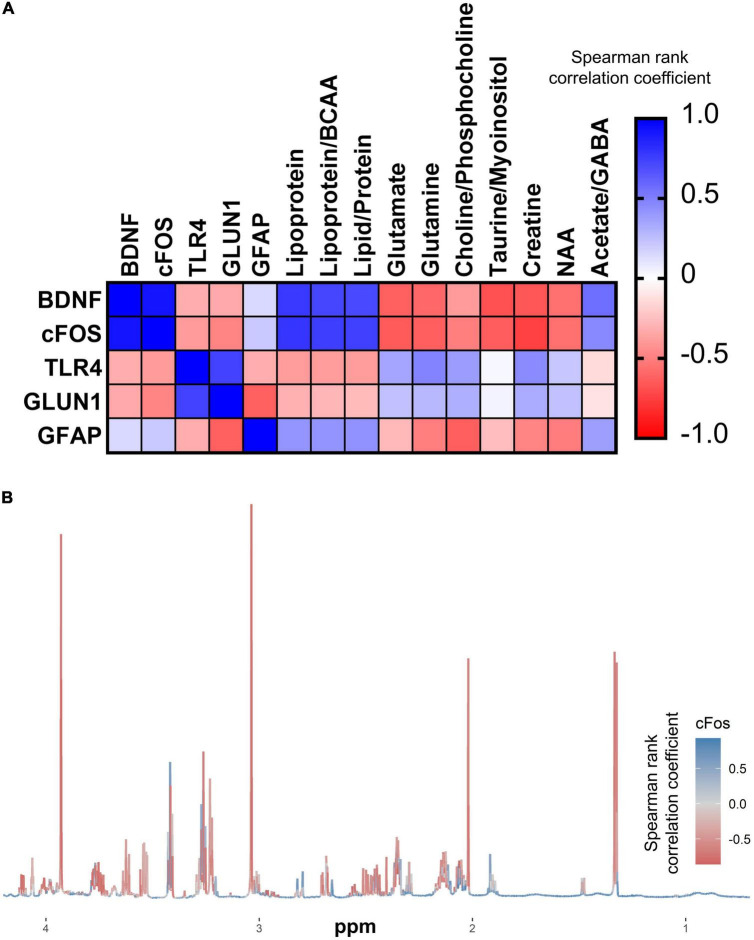
cFOS expression is intimately linked to cerebral metabolism. **(A)** Correlation matrix of qPCR gene expression in the PFC with cerebral metabolites that were significantly altered in GF (*n* = 6) vs. SPF (*n* = 6) mice. **(B)** Median NMR spectrum (*n* = 12) of the brain metabolome, colored by correlation with PFC cFOS expression.

Correlations between gene expression and key brain and liver metabolites were analyzed to infer potential connections between metabolic changes and neuroplasticity. A heatmap was used to visualize correlations (Figure 8); those that remained significant following FDR correction are shown in Supplementary Figure 2. Most notably, *cFOS* expression correlated significantly with several brain metabolites that were also significantly altered by the GF phenotype, whereas only liver succinate was significantly correlated with PFC *cFOS* expression (Figure 8 and Supplementary Figure 3).

## Discussion

In this study, we aimed to define key intermediary factors (metabolites) involved in gut microbiota to brain communication, with implications for the role of the MGB axis in anxiety and other mood disorders (Table 2). We inferred altered brain function from significant changes to brain gene expression between age and strain -matched GF and SPF mice. We report significant gene expression and metabolomic changes in GF compared to SPF juvenile mice in liver and brain tissue. Of particular note, we identified a significant reduction in *cFOS* expression in the prefrontal cortex of GF mice, which may relate to the robust reduced-anxiety phenotype demonstrated by GF animals (Neufeld et al., 2011; De Palma et al., 2015). Furthermore, our results suggest a direct relationship between the microbiota regulation of host metabolism and brain plasticity, as *cFOS* expression correlated with a number of brain metabolites that were altered between GF and SPF animals. These findings shed light on the complex mechanisms underlying the MGB axis and provide insights into potential avenues for the development of novel treatments for stress-related psychiatric disorders such as anxiety.

### Liver metabolism

Microbiota contribute to normal host metabolism. In the GF liver, we identified significantly higher levels of several amino acids usually metabolized by commensals, including leucine, glycine, and phenylalanine. Similarly, lower levels of metabolic by-products were observed, such as succinate and trimethylamine N-oxide (TMAO). These findings are consistent with several reports comparing liver (Mardinoglu et al., 2015) and blood (Lai et al., 2021) metabolomic differences between GF and SPF mice, and likely represent an altered gut amino acid profile (due to the lack of microbial metabolism in GF mice) entering hepatic tissue via the portal vein.

### Liver and brain immunity

Reduced immune markers in the liver (*IL-1ß*, *GFAP*, and *SAA-2*), suggests immunological homeostasis may be disrupted (Furusawa et al., 2013). *GFAP* is expressed on stellate cells in the liver, which are normally thought to be quiescent but may be activated in response to liver injury or inflammation (Winau et al., 2007). It is unsurprising that hepatic *SAA-2* follows a similar expression pattern, as SAA-2 is known to be a primary mediator of the downstream inflammatory response in activated hepatic stellate cells (Siegmund et al., 2016). It is important to note that *SAA-2* may be upregulated several-hundred-fold after an inflammatory challenge (Yates et al., 2022). Thus, a modest 50% reduction in expression may be interpreted as a reduction in basal expression levels, possibly due to a lack of pathogen-associated molecular patterns (PAMPs) entering the portal circulation from the gut.

Although we were initially surprised to find no significant difference in liver *TLR4* expression between GF and SPF mice, this finding has been reported in peripheral tissues by other laboratories (Amaral et al., 2008). It is possible that *TLR4* is constitutively expressed in the liver, and therefore, the discrepancy between GF and SPF mice lies in the level of *TLR4* activation and downstream inflammatory signaling. Our results showed reduced expression of *IL-1B, SAA-2*, and *GFAP* in GF mice relative to SPF mice, suggesting that the absence of circulating microbial antigens reduces basal inflammatory tone in the periphery.

Our study revealed a novel finding of increased *TLR4* expression in the brains of GF mice. We propose that the absence of exposure of brain endothelial cells to circulating PAMPs, such as endotoxin, may have led to an adaptive increase in *TLR4* expression to maintain homeostasis. Alternatively, previous research has shown that microbiota-derived acetate contributes to microglia homeostasis in the rodent brain (Erny et al., 2021). Since we identified a similar reduction in brain acetate levels in GF mice, it is possible that aberrant TLR4 expression could be due to disrupted microglia function, which has not been previously reported.

TLR4 has been proposed to play a significant role in modulating synaptic plasticity during neurodevelopment (Okun et al., 2011), with the exact role of neuronal *TLR4* expression yet to be fully understood. Previous studies have suggested an interaction between *TLR4* expression and NMDAR activity (Kashima and Grueter, 2017). Notably, *TLR4*-deficient animals exhibit deficits in drug reward learning, indicating that microbiota-induced alterations in *TLR4* expression during key neurodevelopmental periods may influence anhedonia through changes in synaptic physiology, specifically NMDAR levels.

### Alterations in synaptic plasticity

We found reduced cortical expression of the synaptogenesis plasticity protein, brain-derived neurotrophic factor (*BDNF*) in GF mice, corroborated by numerous studies examining expression in the cortex, but also other brain regions, including the hippocampus and amygdala (Diaz Heijtz et al., 2011; Neufeld et al., 2011; Clarke et al., 2013). Chronic stress negatively impacts BDNF expression, via glucocorticoid mechanisms (Chen et al., 2017) and so altered development of the hypothalamic pituitary adrenal (HPA) axis in GF mice may explain aberrant *BDNF* expression, though further studies assessing behavioral and physiological responses to stress are required (Sudo et al., 2004). Depression is also associated with a decrease in BDNF, demonstrated by post-mortem analysis in the PFC of depressed individuals (Karege et al., 2005) and in animal models of depression (Li et al., 2016). Indeed, a prevailing theory of depression pathophysiology is a reduced capacity to neurochemically adapt to stress through changes in plasticity (Liu et al., 2017). Disruption of early life commensal-dependent HPA axis development may contribute to depression through BDNF suppression. Disruptions to normal microbiota engraftment in early life due to caesarean birth (Dominguez-Bello et al., 2010) or antibiotic treatment (Fouhy et al., 2012) may therefore have long-term consequences for brain function and increase the risk of stress-related psychiatric conditions.

### The microbiome affects the levels of short-chain fatty acids

Despite considerable debate about the exact microbiota composition alterations in MDD, it is consistently found that bacteria responsible for producing SCFAs (namely, butyrate and acetate), such as *Faecalibacterium*, *Lachnospiraceae*, and *Lactobacillus* are reduced in chronically stressed and depressive individuals (Jiang et al., 2015; Aizawa et al., 2019). Here, we identified reduced brain acetate in GF mice relative to SPF controls. Acetate in particular appears to be crucial for the maintenance of microglial complexity suggesting that continuous microbial input and diversity is continuously required for normal function (Erny et al., 2021). The involvement of microglia in synaptic pruning and developmental neural circuit shaping may contribute to the regulation of stress- and depression-related circuitry in adulthood, further exemplifying how developmental dysbiosis may promote future stress-related depression (Schafer et al., 2012).

Short-chain fatty acids contribution to healthy brain function illuminates a putative therapeutic target. Huang et al. (2021) found acetate supplementation promoted hippocampal plasticity and induced antidepressant-like effects in a chronic social defeat stress (CSDS) mouse model. While this study has exciting potential, it is limited in that it only looked at the positive control, fluoxetine, in terms of behavior not gene expression; it may have been useful to assess if antidepressants produced similar gene expression changes to acetate, possibly indicating similar underlying mechanisms. Nonetheless, since acetate (in the form of glyceryl triacetate) has already been found safe for human use (Madhavarao et al., 2009), it paves the way for clinical human trials to explore its therapeutic potential in stress-related psychiatric disorders.

### Exploring correlations between gene expression and metabolomic profiles

*cFOS* expression in the prefrontal cortex was significantly correlated with the abundance of several metabolites in the cerebrum associated with the GF phenotype. cFOS protein dime rises with members of the Jun family to form activator protein-1 (AP-1), a transcription factor with widespread activating effects on gene expression, such as on neurotransmitter-producing enzymes, glutamate decarboxylase (producing GABA) and tyrosine hydroxylase (producing dopamine) (Najlerahim et al., 1991; Sabban et al., 1995). Alterations in cFOS activity could disrupt the brain’s excitatory-inhibitory balance or dopaminergic modulation, potentially with consequences on anxiety-like behaviors. Given the role of cFOS role as a master regulator, our data putatively suggests an avenue in which microbiota, via modulating the metabolomic landscape, may regulate host brain plasticity.

It is currently unknown which metabolites, and by what mechanism, may regulate cFOS expression, but our findings raise some suggestions. Previous data reveal taurine administration to have a suppressive effect on peripheral cFOS expression in mesenchymal cells (Terashima et al., 2003). Although not yet demonstrated in the brain, there may be overlap in peripheral and central taurine actions which would explain our negative correlation between taurine and cFOS. The observed significant increase in extracellular glutamate in GF mice could result in calcium-mediated neuronal excitotoxic death, reducing the amount of cortical neuronal activity, reflected by reduced cFOS, given its role as a marker of neural activity. A microglial culture study found glutamate receptor activation induced cFOS expression initially, but high concentrations dramatically decreased cFOS expression, supporting our hypothesis (Eun et al., 2004). Preliminary findings also implicate acetate: CSDS-induced depression results in suppressed cFOS expression which can be mostly restored by acetate supplementation (Huang et al., 2021). Given the high level of correlational significance with cFOS and altered metabolites, it warrants further study to determine if a causal link between microbially-affected metabolite levels and cFOS expression exists. This may suggest another potential mechanism of microbial involvement in mood and potentially dysbiosis’ contribution to stress-related psychiatric disorders.

### Future directions

Several further research avenues can be explored from this finding. Firstly, a panel of behavioral assays, addressing anxiety-like behavior, depressive-like behavior, anhedonia, and memory, could be employed to determine how altered *cFOS* expression and brain metabolite levels affect emotional and cognitive behaviors. While several studies have identified reduced anxiety-like behavior in male and female GF mice in adulthood (Luczynski et al., 2016), it would also be important to determine whether this phenotype exists in adolescent mice. Additionally, gene expression in other brain regions (amygdala and hippocampus) could be mapped to the cortex metabolome and other distinct brain regions (brainstem and cerebellum) to more comprehensively investigate gene-metabolite associations. This approach, however, would necessitate mass spectrometry techniques or pooled sampling to overcome the issue of limited tissue mass per brain region.

Electrophysiology experiments could also be employed to understand how changes in brain gene expression and metabolite levels correspond to synaptic changes between brain regions, which may be of particular interest given the observed significant/trending reduction in PFC *cFOS/BDNF*, respectively, and significant/trending increase in *TLR4/GLUN1*, respectively. Specifically, neuronal activity could be investigated in depression-related PFCbrainstem projections (Warden et al., 2012) or midbrainamygdala projections (Chaudhury et al., 2013; Koo et al., 2019; Morel et al., 2022).

Such experiments could be followed-up by the reintroduction of individual commensal strains into GF mice, observing metabolomic and neuronal gene expression changes, and the impact on depressive and anxiety-like behaviors. Mapping neurochemical and behavioral changes to individual commensal strains will progress the MGB axis field toward the development of novel therapeutics, and further elucidate the microbiota’s role in brain metabolism.

### Limitations

Whilst this study provided significant evidence for the impact of the gut microbiome on the host, there are important limitations. Firstly, it may be useful to confirm whether changes in mRNA expression levels reflect changes in protein abundance, particularly of FOS protein. Nevertheless, it has been suggested that *cFOS* transcription, not translation, serves as a readout for neuronal activity in the PFC, and by extension, neuronal plasticity (Sheng et al., 1990). Indeed, this is supported by the significant correlation between *cFOS* mRNA (upstream of FOS protein translation) and cerebral metabolite levels (downstream of FOS protein translation).

Secondly, only female GF and SPF mice were used to assess the gene expression and metabolomic correlations in this study. While the use of female mice is justified by the increased prevalence of disorders of gut-brain interaction, such as irritable bowel syndrome in females (Canavan et al., 2014), we recommend that further research explores whether sex differences may exist in the GF phenotype on PFC gene expression and metabolite levels. In this study, we performed qPCR on prefrontal cortex tissue and metabolomics on the contralateral cerebrum. Therefore, the correlation analysis compares different brain regions, which may limit the conclusions drawn from these results. Conversely, comprehensive metabolomic studies of the mouse brain across the lifespan have shown that in “adolescence” (3 weeks), the cerebrum exhibits a high degree of correlation between distinct brain regions (Ding et al., 2021). Whilst differences in the aforementioned study were noted between the cerebrum as a whole, the brainstem, and cerebellum, and between cerebral regions in adult and aged mice, the high degree of convergence within the adolescent cerebrum is most pertinent to our current study. Importantly, the paper utilized 3-week-old, female, C57Bl/6 mice, identical to our own study. Separately, we have demonstrated similar findings, whereby cerebrum metabolite levels and PFC mRNA correlations are stronger in 3-week-old mice compared to 16-week-old mice (Radford-Smith et al., 2022). Therefore, it is unlikely that performing metabolomics on just the contralateral PFC region would show different results to the metabolomics and correlation analyses performed here.

## Conclusion

This study demonstrated that microbiota clearly exert a significant impact on host physiology. Until recently, their effect on brain function was largely underestimated. This study observed gene expression and metabolomic changes, identifying novel correlations between cFOS expression and several metabolites. Our pilot study provides preliminary evidence that the host metabolome profile, modified by the microbial composition, may influence brain gene expression, at least partially through regulation of the widespread transcriptional regulator, cFOS and putatively through HPA axis regulation. The microbiome’s impact on metabolite levels and gene expression, may be crucially implicated in depression, through neuroinflammatory, and neuroplasticity mechanisms. It is therefore not surprising that dysbiosis, even in early life, is strongly implicated in stress-related psychiatric disorders. The gut microbiome and associated metabolites, such as SCFAs, may be an important therapeutic target for novel or augmenting treatment strategies for those afflicted with depression.

## Data availability statement

The raw data supporting the conclusions of this article will be made available by the authors, without undue reservation. The liver and brain metabolomics data analyzed in this study is available online at http://dx.doi.org/10.5287/ora-zv6mnkajn.

## Ethics statement

This animal study was reviewed and approved by the UK Animal Welfare and Ethical Review Body. All animal procedures were carried out in accordance with UK Home Office Animals (Scientific Procedures) Act (1986) and associated Home Office guidelines under license P996B4A4E.

## Author contributions

TP and DR-S performed the research and conducted the analysis. TP, DR-S, and DA drafted the manuscript. All authors contributed to the article and approved the submitted version.
