# Supplementary material for: cFOS expression in the prefrontal cortex correlates with altered cerebral metabolism in developing germ-free mice
Source: Front Mol Neurosci. 2023 Apr 20;16:1155620. doi: 10.3389/fnmol.2023.1155620 (PMC10157641; doi:10.3389/fnmol.2023.1155620)
Supplement: Supplementary file 1 [file Data_Sheet_1.docx]

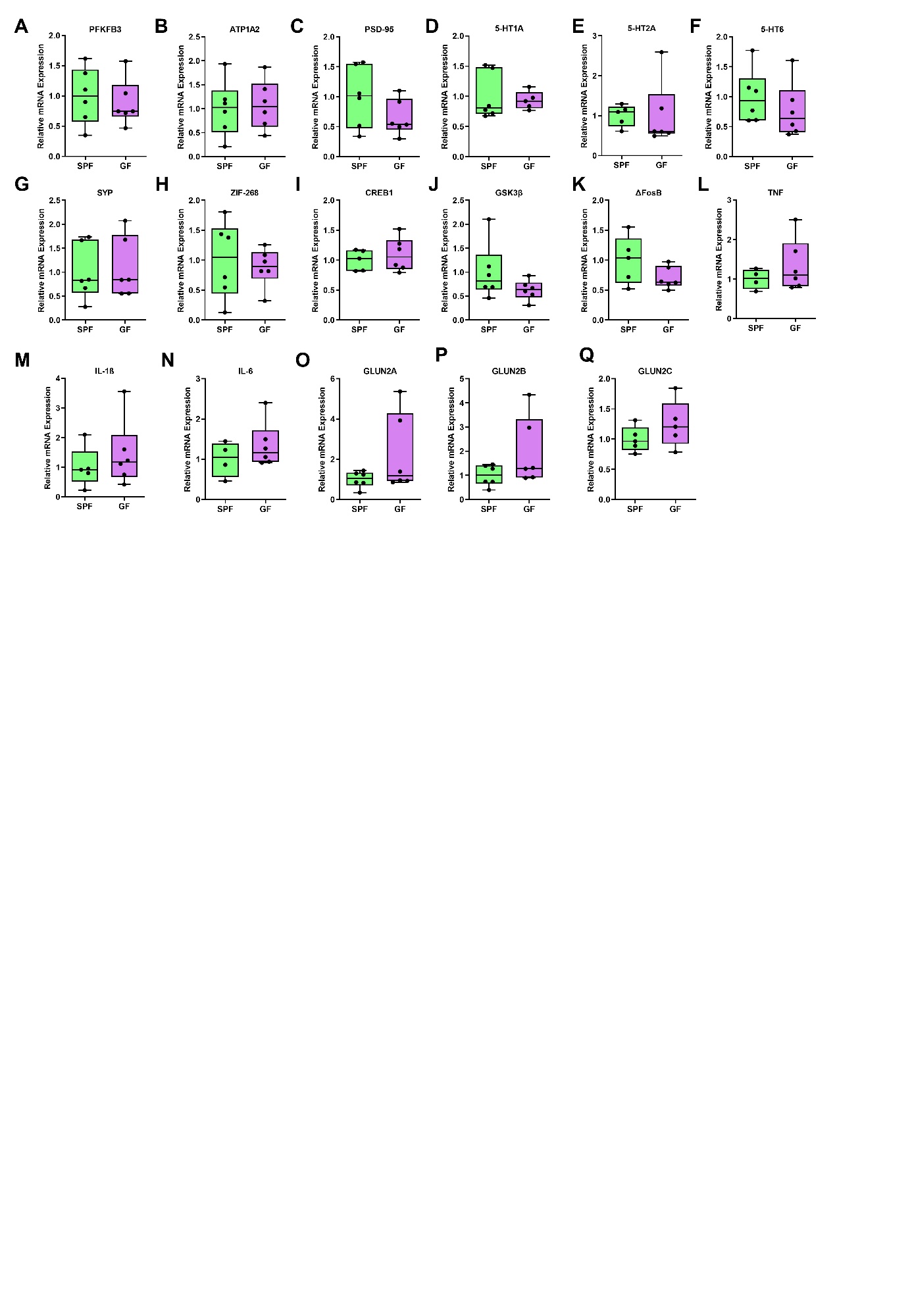
**Figure S1. Non-significant genes from qPCR analysis of prefrontal cortex.** Boxplots represent the interquartile range (IQR) and minimum/maximum values. There were no significant differences in gene expression for the following genes. **(A)** PFKFB3 (p=0.6464). **(B)** ATP1A2 (p=0.8041). **(C)** PSD-95 (p=0.1782). **(D)** 5-HT1A (p=0.4740). **(E)** 5-HT2A (p=0.9867). **(F)** 5-HT6 (p=0.4015). **(G)** SYP (p=0.8029). **(H)** ZIF-268 (0.6848). **(I)** CREB1 (p=0.5260). **(J)** GSK3ß (p=0.1748). **(K)** ΔFosB (p=0.1370). **(L)** TNF (p=0.55619). **(M)** IL-1ß (p=0.4554). **(N)** IL-6 (p=0.3333). **(O)** GLUN2A (p=0.1553). **(P)** GLUN2B (p=0.1634). **(Q)** GLUN2C (p=0.2506).


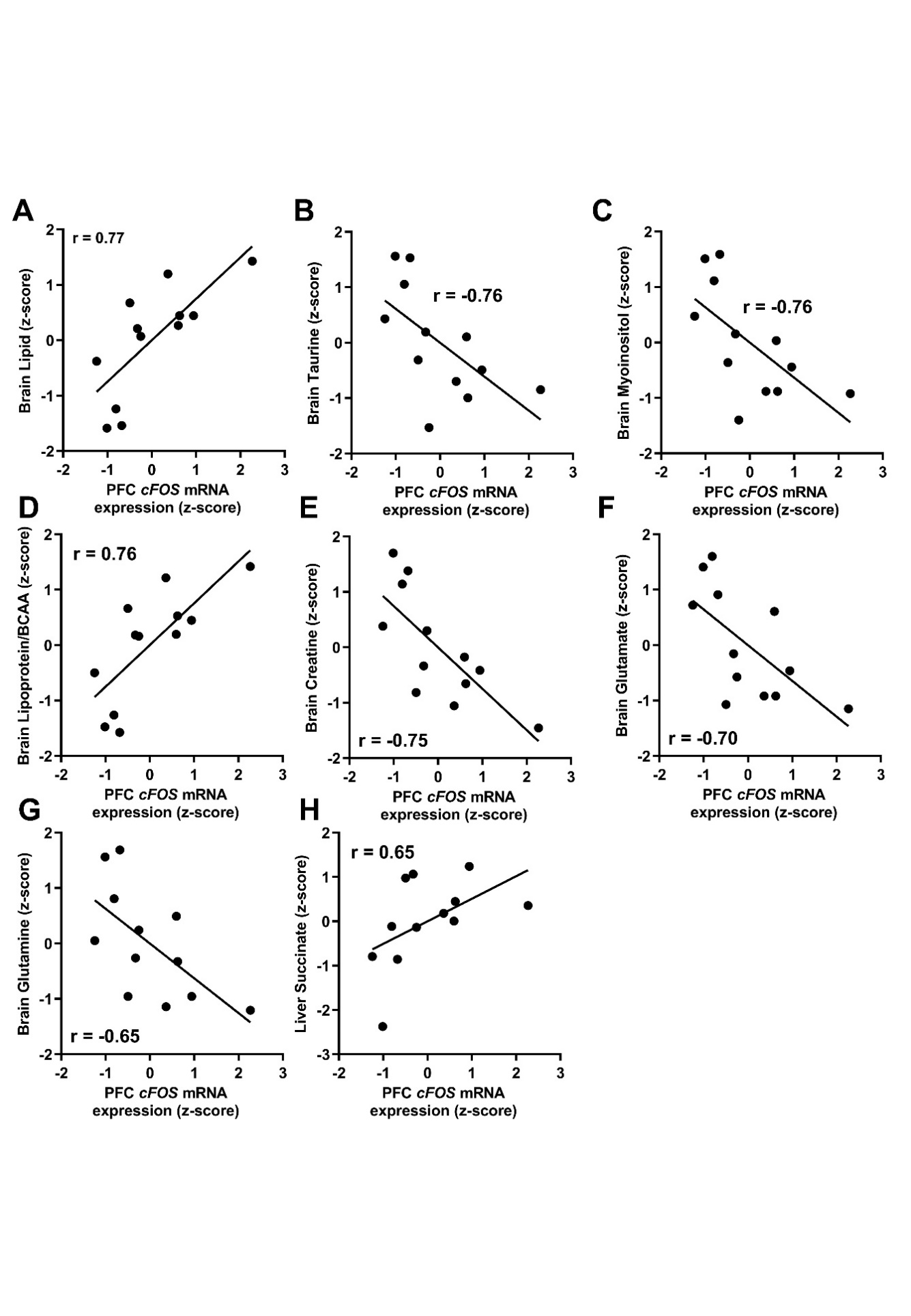


**Figure S2. Univariate correlation analysis between prefrontal cortex cFOS expression and key cerebral metabolites between GF (n=6) and SPF (n=6) mice.** All correlations were calculated using Spearman’s rank analysis and multiple testing was accounted for using the Benjamini and Hochberg correction for multiple testing (q<0.05). Significant correlations between cFOS gene expression and metabolites. **(A)** Brain lipid levels were positively correlated with cFOS (r = 0.77; q = 0.013451), **(B)** brain taurine levels were negatively correlated (r = -0.76; q = 0.013451), **(C)** brain myoinositol levels were negatively correlated (r = -0.76; q = 0.013451), **(D)** brain Lipoprotein/BCAA levels were positively correlated (r = 0.76; q = 0.13451), **(E)** brain creatine levels were negatively correlated (r = -0.75; q = 0.013451), **(F)** brain glutamate levels were negatively correlated (r = -0.70; q = 0.025132), **(G)** brain glutamine levels were negatively correlated (r = -0.65; q = 0.038560), and **(H)** liver succinate levels were positive correlated (r = 0.65, q = 0.038560).


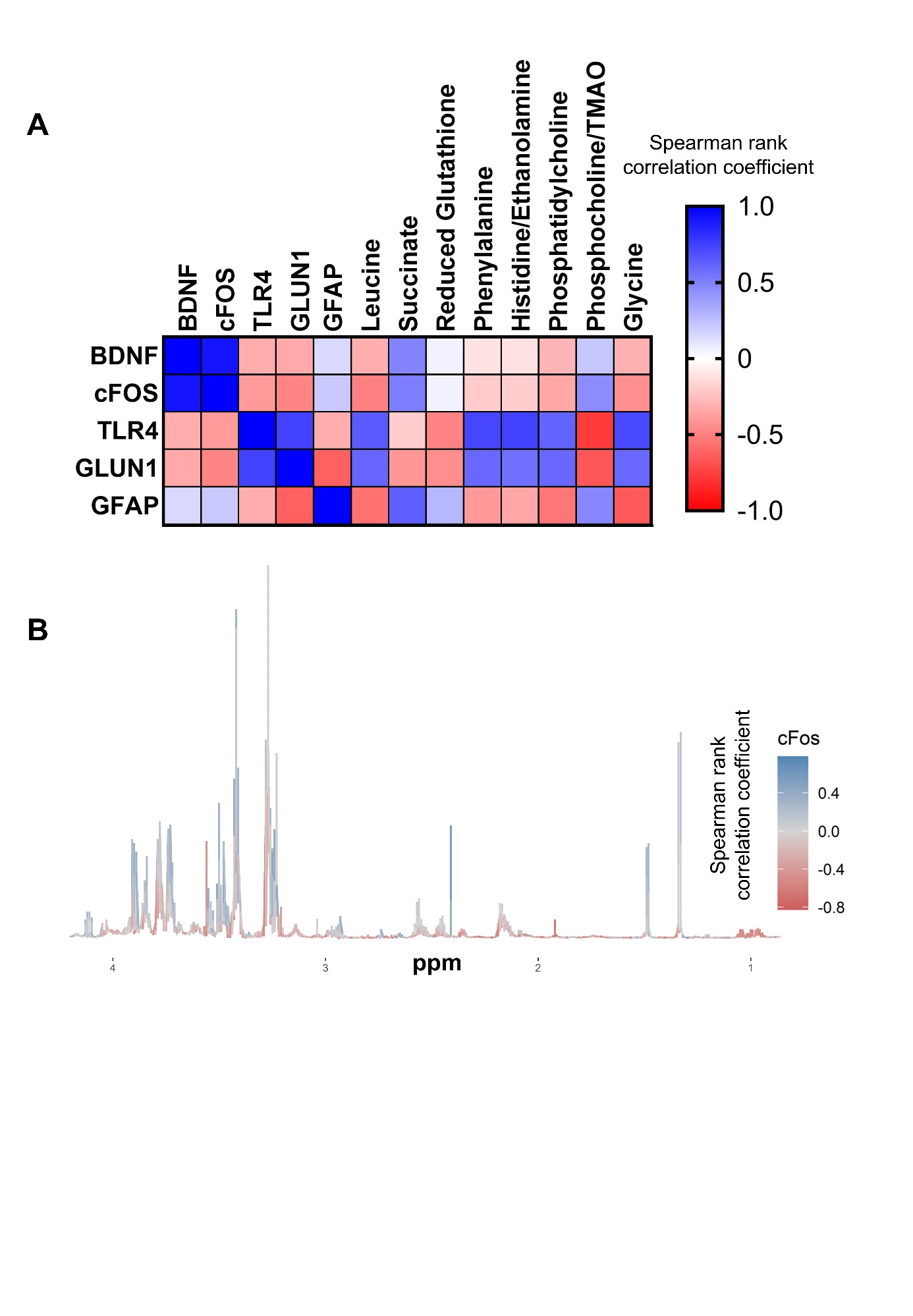
**Figure S3. cFos expression in the PFC is not correlated with the liver metabolome. (A)** Spearman correlation matrix between key PFC genes and key liver metabolites of GF (n=6) and SPF (n=6) mice, and **(B)** the median NMR spectra of the liver metabolome coloured by its correlation with PFC *cFos* mRNA expression.
